# Multiple recurrent myxofibrosarcoma of the orbit: case report and review of the literature

**DOI:** 10.1186/s12886-020-01458-1

**Published:** 2020-07-06

**Authors:** Baixue Du, Xin He, Yujiao Wang, Weimin He

**Affiliations:** 1 Department of Ophthalmology, West China Hospital of Sichuan University, No. 37 Guoxue Xiang, Wuhou District, Chengdu, 610041 Sichuan Province China; 2 Department of pathology, West China Hospital of Sichuan University, No. 37 Guoxue Xiang, Wuhou District, Chengdu, 610041 Sichuan Province China

**Keywords:** Orbit, Multiple myxofibrosarcoma, Recurrent myxofibrosarcoma

## Abstract

**Background:**

Although myxofibrosarcoma (MFS) is the most common mesenchymal tumor, occurrence in the orbit is extremely rare. A characteristic clinical feature of MFS is its propensity for local recurrence (LR). Still, none of published literature has described the entire clinical course of multiple recurrences of MFS in the orbit. Here we present an unusual case in which a patient with multiple recurrences of MFS in the orbit followed-up for 5 years. We describe its clinical-pathological correlation, treatment, and prognosis.

**Case presentation:**

A 70-year-old woman sought treatment for a year history of right upper eyelid swelling in May 2014. Since then, she underwent three surgical procedures in the right orbit in the same region (in 2014, 2016, and 2017). The pathology analysis of the three surgical tissue samples was consistent with myxofibrosarcoma, characterized by spindle-shaped cells and variably myxoid stroma. In the 2 years follow-up after the third surgery, there was no evidence of tumor recurrence.

**Conclusion:**

Myxofibrosarcoma of the orbit is extremely rare. Since few reports are available in the literature, the diagnosis and management of the orbit myxofibrosarcoma is still a challenge to ophthalmologists. Histopathology is highly valuable in the diagnosis. As a clinical characteristic, the lesions have a high risk of local recurrence; therefore, aggressive resection and careful postoperative follow-up are paramount.

## Background

Myxofibrosarcoma (MFS) is the most common malignant soft tumor of the elderly, previously known as myxoid malignant fibrous histiocytoma (MFH) [1]. Since the new 2002 tumor classification system published by the World Health Organization (WHO), these tumors are considered as distinct pathological entities [2]. MFS usually appears in the body extremities, especially lower extremities, then trunk. Occurence in the orbit is extremely infrequent. In this article we describe a 70-year-old woman with a myxofibrosarcoma afflicting the orbit, presenting with multiple local recurrences.

## Case presentation

A 70-year-old woman presented to our hospital with a one-year history of right upper eyelid swelling in May 2014. Her best-corrected visual acuities (BCVAs) were 0.6 in the right eye, 1.0 in the left eye. Binocular Intraocular pressure was normal. Physical examination revealed a palpable soft mass in the right upper eyelid with a size of 3 cm*2 cm. The exams of the right upper eyelid also presented with an increased volume. Complete resection of the tumor revealed a large oval encapsulated mass in close relation to the adjacent tissue. The histopathology examination revealed a spindle-cell tumor with a myxoid matrix, consistent with MFS. The Ki-67 proliferation index was 60%.

Two years after the initial surgery, the patient was admitted to another hospital with a palpable mass at the same site and swollen right upper eyelid. She underwent surgery and pathological analysis confirmed the recurrence of MFS, with an invasion of the surrounding tissue. Immunohistochemical staining was negative for S-100, desmin, and positive for α-SMA and for Ki67 in 20% of nuclei. Unfortunately, no pathological section was available. Adjuvant radiation therapy was administered after surgery, but we were unable to collect information about the radiation therapy modalities.

**Fig. 1:**
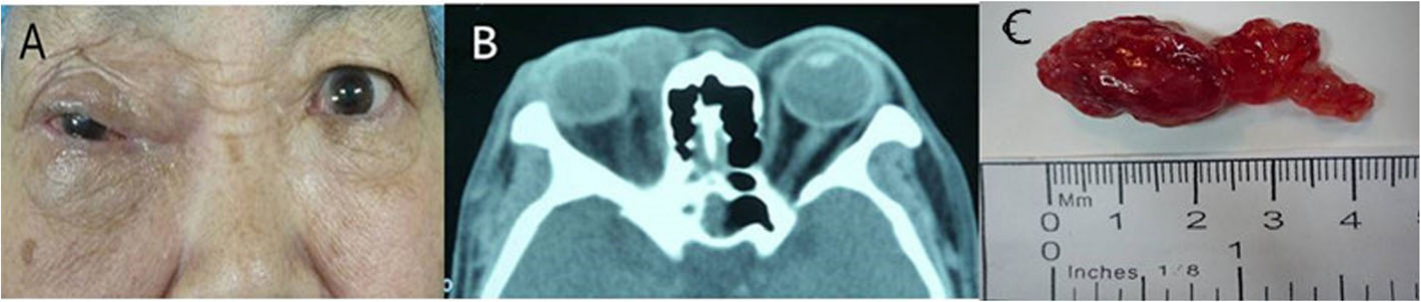
**a** Photograph showing swelling and ptosis of the right eyelid. **b** Contrast-enhanced orbital CT showing a well-defined mass which compressed the lateral eyeball. **c** Gross appearance of the tumor showing a lobulated-shaped mass with local necrosis

**Fig. 2:**
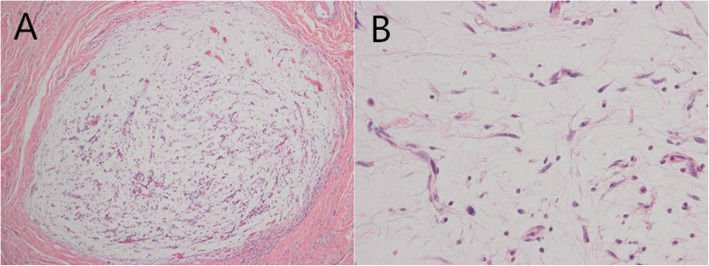
**a** At low power, the tumor shows nodular growth with a prominent myxoid matrix (hematoxylin-eosin, × 100). **b** At high power the tumor shows spindle-shaped cells and elongated, curvilinear, thin-walled blood vessels (hematoxylin-eosin, × 400)

**Fig. 3:**
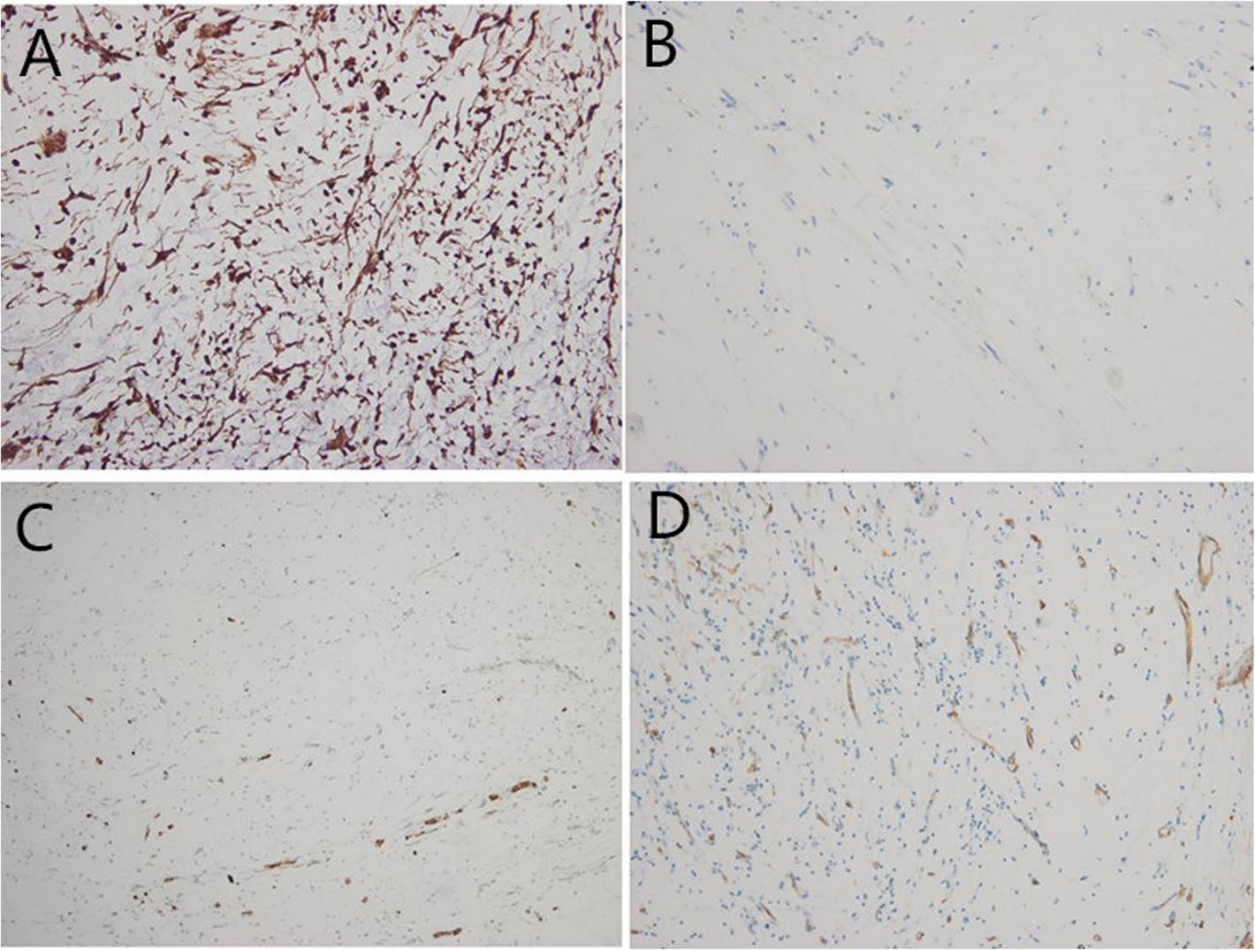
Immunohistochemical analysis of the tumor tissue was positive for Vimentin (**a**) but negative for desmin (**b**) and S-100 (**c**). CD 34 was partially positive (**d**)

One year after the second tumor resection, she returned to our hospital with progressive swelling of the right eyelid for nearly half a year. Ophthalmological examination showed visual acuity as CF/40 cm OD and 0.6 OS. The upper eyelid was swollen and ptotic. There was a 1.5 cm*1.5 cm palpable, firm, and movable mass in the inner part of the right upper eyelid (Fig. 1a). Slit lamp examination of the right eye showed a 6 mm*2 mm ulcer in the temporal cornea and a non-round pupil with extensive posterior synechiae and absent light reflex. The left eye examination revealed no abnormalities. Contrast-enhanced orbital CT showed a well-defined cystic to solid mass with a size of 1.9 cm *1.9 cm in the superolateral part of the orbit, compressing the lateral eyeball (Fig. 1b). The patient underwent surgical tumor resection again revealing a lobulated shaped and firm mass with local necrosis (Fig. 1c). The final histological examination confirmed myxofibrosarcoma (MFS). On the microscopic examination it was visualized a nodular growth tumor with a prominent myxoid matrix (Fig. 2a). Another characteristic finding was the presence of prominent, elongated, curvilinear, thin-walled blood vessels (Fig. 2b). Immunohistochemical staining was positive for Vimentin (Fig. 3a), negative for desmin (Fig. 3b), S-100 (Fig. 3c), SMA and EMA. CD 34 was partially positive (Fig. 3d) and the proliferation marker Ki-67 positive rate was less than 1%.

After the third surgery, the patient was followed-up for 2 years without tumor recurrence.

## Discussion and conclusions

Myxofibrosarcoma (MFS) is the most common mesenchymal tumor in elderly patients, which, most of the time, presents as a painless, slowly-growing mass, with typical potential for local recurrence (LR). It is characterized by variably myxoid stroma with a distinct curvilinear vascular pattern.

MFS might develop in most parts of the body, but most commonly in the extremities, especially lower extremities, then the trunk. Orbital presentation of MFS is extraordinarily rare, and it was first described in 2008. Since then, only six cases have been published [3–8].

MFS has a high incidence of LR, ranging from 50 to 60%. Some studies demonstrate locally recurrent MFS progressing to a higher-grade disease with an associated increase in metastatic potential [9, 10]. However, none of these case series studies included orbital MFS. In the six published case reports featuring orbital MFS, the most extended follow-up period was 2 years, which cannot properly reflect relapsing or metastasis. One article has reported a case in which the patient underwent three surgeries as well [7]. Still, no pathological data was described regarding the former operations, making it hard to determine if it was a case of recurrent MFS. In our case we reported a 5 years follow-up after the initial surgery. The entire clinical course with two relapses can demonstrate the aggressive growth pattern of orbital MFS.

The diagnosis of MFS relies on the histopathology. Typically, the spindle-cells or stellate shaped cells populate in variably myxoid stroma and there are great differences in cellularity, polymorphism, and mitotic activity. A curvilinear vascular pattern can be remarkably found in most tumors [2, 11, 12]. Immunohistochemically, the tumor cells may show strong staining with vimentin and would be negative for Desmin, S-100, SMA [2, 11]. The histopathological differential diagnosis of MFS includes benign and malignant lesions. Benign lesions, such as nodular fascitis, myxoma, and spindle-cell lipoma, are all lack extensive vasculature. Malignant lesions include myxoid liposarcoma, pleomorphic dermal sarcoma, myxoid dermatofibrosarcoma protuberans, and low-grade fibromyxoid sarcoma. One of the most difficult distinctions is the low-grade fibromyxoid sarcoma, which occurs mostly in young patients and is characterized by contrasting fibrous and myxoid areas, spindle-cells in a swirling and whorled pattern, with a variably myxoid [1, 13, 14].

Since this kind of tumor is apt to recurrence and metastasis, margin-negative surgical resection is the most effective therapy. Insufficient tumor-free margins are associated with a high risk of local recurrence and, hence, a poor prognosis [15, 16]. The role of radiation therapy and chemotherapy on the treatment of primary MFS remains uncertain. Nonetheless, some studies showed adjuvant radiation therapy providing the best local control [16–18]. In present case, although radiotherapy was used in second treatment, the period of recurrence is shorter than the surgery in our hospital, maybe suggest that the adjuvant radiation therapy provide local control effectively limited.

Summarizing, primary MFS of the orbit is rare and can present a high recurrence rate. Our report described an entire clinical course of multiple recurrences of orbital MFS, reinforcing the importance of complete excision of these lesions. Ophthalmologists should be mindful of this diagnosis and closely monitor these patients.

## Data Availability

All data and supplementary information are included in this published article.

## Abbreviations

MFS: Myxofibrosarcoma
MFH: Myxoid malignant fibrous histiocytoma
BCVA: Best corrected visual acuity
LR: Local recurrence
